# Modulating Effect of the −158 ^G^γ (C→T) X*mn*1 Polymorphism in Indian Sickle Cell Patients

**DOI:** 10.4084/MJHID.2012.001

**Published:** 2012-01-15

**Authors:** Sanjay Pandey, Sweta Pandey, Rahasya Mani Mishra, Renu Saxena

**Affiliations:** 1 Department of Hematology, AIIMS, New Delhi, India; 2 Department of Environmental Biology, APS University Rewa, India

## Abstract

X*mn*1 polymorphism is a known factor, which increases fetal haemoglobin production. Among the inherited disorders of blood, thalassaemia and Sickle Cell Diseases contributes to a major bulk of genetic diseases in India. Our aim was to verify the role of the X*mn*1 polymorphism as a modulating factor in sickle cell patients and frequency of the polymorphism in Indian sickle cell patients. 60 sickle homozygous and 75 sickle beta thalassemia patients were included and 5 ml blood sample was collected from them. Screening of sickle patients was done by HPLC. An automated cell analyzer SYSMEX (K-4500 Model) was used to analyze the Complete Blood Count of patients. X*mn*1 polymorphism analysis was done by PCR-RFLP and one-way ANOVA test was applied to analysis of variance between groups. Among the sickle patients 27 were heterozygous (+/−) and 19 were homozygous (+/+) while 30 were heterozygous (+/−) and 24 were homozygous (+/+) in sickle β-thalassemia patients. Extremely significant differences (p-value <0.001) of hematological parameters seen among patients with Xmn1 carrier and without the X*mn*1 carrier. In our cases the clinical symptoms were barely visible and higher HbF level with X*mn*1 carriers were found. Presence of X*mn*1 polymorphism in sickle cell patients with higher HbF were phenotypically distinguished in the sickle cell patients. We conclude that the phenotypes of Indian sickle cell patients were greatly influenced by X*mn*1polymorphism.

## Introduction

The C-T substitution at position −158 of the Gy globin gene, referred to as the Xmn1-γ polymorphism, is a common sequence variant in all population groups, present at a frequency of 0.32 to 0.35.^1^ Clinical studies have shown that under conditions of hematopoietic stress, for example in homozygous β-thalassemia and sickle cell disease, the presence of the Xmn1-Gγ site favors a higher Hb F response. This could explain why the same mutations on different β chromosomal backgrounds are associated with disease of different clinical severity.^2^,^3^ Increased levels of fetal hemoglobin (Hb F or a2 γ2) are of no consequence in healthy adults, but confer major clinical benefits in patients with sickle cell anemia (SCA) and β-thalassemia, diseases that represent major public health problems.^4^ Fetal hemoglobin (Hb F or a2 γ2) is predominant in red cells of the fetus and the newborn baby, and is largely replaced after birth by adult hemoglobin (α2β2). The two types of γ chains of Hb F (Gγ and Aγ) differ at position 136 (glycine versus alanine) and are produced by closely-linked genes of the β-globin gene cluster. In normal adults, red cells have less than 1% Hb F, and Gγ accounts for some 40% of total γ chain.^5^ Genetic variation of Gγ values has been observed in sickle cell anemia (SS) patients, whose increased Hb F levels facilitate such studies. Although most have 40% Gγ, some have Gγ values of 60% to 70%.^6^ About 5% of black sickle cell patients are heterozygous for the normal -Gγ-Aγ and a mutant -Gγ-Aγ chromosome with both genes producing Gγ globin; they have Gγ values of about 70%.^7^–^9^ Approximately 1/6 black sickle cell and 2/3 black β-thalassemia heterozygotes have high Gγ values of about 60%. It has been estimated that in India with a population of 100 million at the millennium (2000) and a birth rate of 25/1000, there would be about 45 million carriers and about 9000 infants born each year with haemoglobinopathies.^10^ Among the genetic factors known to affect HbF production are DNA sequence variations within the β-globin gene cluster. In particular, the (C-T) variation at position − 158 upstream of the Gγ globin gene, which is detectable by the restriction enzyme X*mn*1. The sequence variation has been shown to increase Hb F levels in β-thalassaemia anemia.^11^,^12^,^13^ There is a paucity of data for the effect of the X*mn*1 polymorphism on the phenotype of Indian sickle cell patient; thus our aim was to evaluate the role of the X*mn*1 polymorphism as a modulating factor in sickle cell patients and its frequency.

## Material and Methods

Subjects were 60 sickles homozygous and 75 sickle beta thalassemia patients (29 patients were HbSβ^+^ while 46 patients were HbSβ^0^). About 5 ml blood sample was collected from hematology outpatient department AIIMS; after taking their consent. Study was approved from institutional ethical committee. Clinical evaluation was done during physical examination (visually appeared) as well as laboratory evaluation. Age of onset of splenomegaly and jaundice was <10 year and the presence of anemia was evaluated through hemogram analysis. Complete blood count and red cell indices were measured by automated cell analyzer (SYSMEX K-4500, Kobe Japan). Quantitative assessment of hemoglobin Hb F, Hb A, Hb A2 and Hb S and diagnosis of HbSS and HbSβ-thalassemia was performed by high performance liquid chromatography (HPLC-Bio-Rad-Variant^TM^ Bio Rad, CA, USA). DNA extraction done by phenol chloroform method and DNA quantification done by nano drop spectrophotometer. X*mn*1 polymorphism analysis was done by PCR-RFLP method as per Sutton^14^ et.al (1989). A one-way ANOVA test was applied to analysis of variance between groups. P-value <0.05 were considered statistical significant. Presence of clinical symptoms with and without X*mn*1polymorphism was used to for comparison of clinical features.

## Result

Sixty sickle homozygous (35 male and 25 female with mean age 11.32±7.61 years) and 75 sickle β-thalassemia (57 male and 18 female with mean age 12±8.33 years) patients were characterized. Out of 60 sickle homozygous patients; 27 (45%) were heterozygous (+/−) and 19 (31.67%) were homozygous (+/+) while 30(40%) were heterozygous and 24(32%) were homozygous in sickle β-thalassemia for X*mn*1 polymorphism. Fourteen (23.33%) patient in sickle homozygous and 21(28%) patients in sickle β-thalassemia were normal for X*mn*1 polymorphism. The frequency of the X*mn*1 polymorphism was higher among sickle homozygous than sickle β-thalassemia patients. Clinical severity were improved with homozygous (+/+) X*mn*1 polymorphism in sickle cell anemia as well as sickle β-thalassemia patients. Reticulocytes, haemoglobin and red cell indices were higher in X*mn*1carriers than non carriers and found extremely statistically significant (p-value <0.001). The explanation to the improvement of RBCs in X*mn*1 carriers is that the anemia was less and overall red cell indices and Hb value was improved. Hyperpigmentation was found in a few cases. Splenomegaly, gall stone, painful crisis, jaundice and frequency of blood transfusion in relation with HbF and X*mn*1 polymorphism were lesser in X*mn*1 carriers in comparison to non-carriers and found statistically significant (p-value <0.001). Details of haematological parameters and clinical parameters of HbSS and HbSβ-thalassemia are given in table 1, 2, 3 and 4 respectively.

## Discussion

Fetal hemoglobin (HbF) genes are genetically regulated and the level of HbF and its distribution among sickle erythrocytes is highly variable. Hb F is the major genetic modulator of the hematological and clinical features of sickle cell disease with the Senegal and Saudi-Indian haplotype which gives its beneficial effects to the pateints.^15^, ^16^ Many epidemiological studies suggested that disease complications most closely linked to sickle vasoocclusion and blood viscosity were robustly related to HbF concentration while complications associated with the intensity of hemolysis were less affected. Although HbF is the protective factor for leg ulcers, one complication closely associated with hyper-hemolysis.^17^,^18^ The effect of −158 C > T mutation on expression of Gγ globin gene has been the subject of considerable interest. The association of some β-globin mutations with X*mn*1 site with elevated HbF expression has been previously published. The role of increased HbF response as an ameliorating factor has become evident in patients who were mildly affected despite being homozygotes or compound heterozygotes for β^0^ or β^+^ thalassaemia.^19^ Association of X*mn*1 and frequency of blood transfusion was reported in thalassemic patients.^20^, ^21^

The strong association of X*mn*1 site with the Arab Indian haplotype is thought to be associated with high fetal hemoglobin concentration and confer a benign course of the disease. However, the clinical presentation of sickle cell disease in different regions in our country is highly variable.^22^ In our cases, hematological parameters of sickle homozygous and sickle β-thalassemia an improved condition with X*mn*1 carriers while non carriers of X*mn*1polymorphism were found worsened. Our cases of sickle cell patient showed the presence of C-T variation at position −158 in the Gγ gene, affect hematologically as well as clinically and increased production of HbF. X*mn*1 carriers of HbSβ-thalassemia patients had higher HbF than HbSS patients. The clinical features of HbS-β thalassemia are extremely variable, ranging from a completely asymptomatic state to a severe disorder similar to homozygous sickle cell disease. This heterogeneity is likely to be due to the presence of different β-thalassemia alleles or interaction with modulating genetic factors like associated α-thalassemia and/or a gene for raised HbF production (X*mn*1 polymorphism).^23^ Heterozygosity for presence of X*mn*1 site polymorphism is also likely to influence phenotype^24^ and X*mn*1 polymorphism absence reduction is associated with acquired HbF elevation.^25^ Gilmans^13^ data are consistent with the hypothesis that T at position - 1 58 causes the high HbF values. There is a paucity of data in relation of X*mn*1polymorphism and phenotypic effect on Indian sickilers. However a study on HbEβ-thalassemia report the phenotypic effect of X*mn*1 polymorphism^17^ while another study report none of the association in clinical severity and presence of X*mn*1 polymorphism in thalassemia intermedia patients.^26^ Raina et al.^27^ concluded that the presence of X*mn*1 polymorphism and IVS 1-1 mutation leads to a milder phenotypic presentation causing a delay in onset of blood transfusions but dose not effect the amount of blood received /kg/year. However α-thalassemia also influence on the level of HbF in patients with sickle cell disease.^28^,^29^ In our cases the frequency of X*mn*1 polymorphism found higher amongst sickle cell anemia patient in comparison to sickle β thalassemia. Sickle homozygous and sickle β-thalassemia patients showed clinical variation and this could be due to the association of Xmn1polymorphism, either in homozygous or heterozygous state. Presence of Xmn1polymorphism in sickle patients with higher HbF that improve phenotypic presentation in the sickle cell patients. A study from Western Iran in β-thalassemia patients report the presence of Xmn1 polymorphic site on both chromosomes (+/+) the level of Hb F tended to be increased compared to the absence of X*mn*1 ( / ) and the presence of this polymorphic site caused a positive influence on Hb F production and the ^G^γ percent which could improve the clinical symptoms of β-thalassemia patients.^30^ Our finding in sickle cell disease patients was similar with the study. Thus we conclude that the phenotypes of Indian sickle cell patients were greatly influenced by X*mn*1polymorphism.

## Figures and Tables

**Table 1:** Comparative hematological parameters of carrier and non carrier Xmn-1 in HbSS.

Mean±SD				

Hematological Parameters	Xmn-1 (+/+) N=19	Xmn-1 (+/−) N=27	Xmn-1 (−/−) N=14	P-value

RBC millions/μl	3.8±2.3	3.2±1.7	3.5±1.2	0.001

HGB g/dl	10.3±1.7	9.3±1.5	9.2±1.2	<0.001

HCT %	20.6±3.4	20.2±1.3	19.8±2.1	<0.001

MCV fl	73.1±5.2	71.3±4.3	71.5±4.7	<0.001

MCH pg	31.6±7.4	30.2±3.2	29.6±3.4	<0.001

MCHC g/dl	32.7±5.2	32.4±3.5	32.6±4.2	<0.001

HbF %	25.2±4.3	20.18±3.7	13.2±4.5	<0.001

**Table 2:** Comparative hematological parameters of carrier and non carrier Xmn-1 in HbSβ-thalassemia.

Mean±SD				

Hematological Parameters	Xmn-1 (+/+) N=24	Xmn-1 (+/−) N=30	Xmn-1 (−/−) N=21	P-value
RBC **millions/μl**	3.7±1.2	3.2±1.7	2.8±1.5	<0.001

HGB **g/dl**	10.8±1.2	9.3±1.7	9.2±1.4	<0.001

HCT **%**	23.7±3.4	23.8±2.6	21.8±2.1	<0.001

MCV **fl**	72.8±6.3	70.9±4.8	68.3±6.5	<0.001

MCH **pg**	34.6±5.4	32.6±4.6	32.8±3.4	<0.001

MCHC **g/dl**	33.5±3.2	30.7±4.2	30.6±3.1	<0.001

HbF **%**	36.2±4.7	32.1±5.2	17.6±4.8	<0.001

**Table 3:** Comparative clinical parameter with carrier and non carrier X*mn*1in HbSS patients.

Frequency %			

Clinical Parameters	Xmn-1 (+/+) N=19	Xmn-1 (+/−) N=27	Xmn-1 (−/−) N=14
Anemia	7 (36.84%)	17 (62.96%)	10 (71.42%)
Hepatomegaly	3 (15.78%)	6 (22.22%)	4(28.57%)
Splenomegaly	2 (10.52%)	5 (18.51%)	4 (28.57%)
Painful crisis	3 (15.78%)	4 (14.81%)	5 (35.71%)
Gall stone	2 (10.52%)	4 (14.81)	5 (21.42%)
Acute chest syndrome	2 (10.52%)	4 (14.81%)	3 (21.42%)
Jaundice	3 (15.78%)	4 (14.81%)	3 (21.42%)
Hyper pigmentation	4 (21.05%)	2 (7.4%)	2 (14.28%)
Avascular necrosis	3 (15.78 %)	2 (7.4%)	2 (14.28%)
Retinopathy	2 (10.52%)	1 (3.7%)	1 (7.14)
Septicemia	1 (5.26%)	2 (7.4%)	None
Deep vein thrombosis	None	1 (3.7%)	None
Pulmonary embolism	1 (5.26%)	None	None
Leg ulcer	1 (5.26% )	None	1(7.14%)
Frequency of blood transfusion	7 (36.84 %)	9 (33.33%)	4 (28.57%)

**Table 4:** Comparative clinical parameter with carrier and non carrier X*mn*1in HbSβ –thalassemia patients.

Frequency %			

Clinical Parameters	Xmn-1 (+/+) N=24	Xmn-1 (+/−) N=30	Xmn-1 (−/−) N=21
Anemia	11 (45.83%)	16 (53.33 %)	14 (66.66 %)
Hepatomegaly	3 (15.78%)	8 (26.66 %)	5 (23.8%)
Splenomegaly	6 (25 %)	9 (30%)	7 (33.34%)
Painful crisis	7 (29.16 %)	9 (30 %)	10 (47.61 %)
Gall stone	6 (25 %)	8 (26.66%)	7 (33.33%)
Acute chest syndrome	5 (20.84%)	7 (23.34%)	7 (33.34)
Jaundice	4 (16.67%)	6 (20%)	5 (23.8%)
Hyper pigmentation	2 (8.33%)	3 (10%)	2 (9.52 %)
Avascular necrosis	1 (4.16%)	3 (10 %)	2(9.52 %)
Retinopathy	None	2 (6.66 %)	1 (4.76 %)
Septicemia	2 (8.33 %)	1 (3.33 %)	None
Deep vein thrombosis	1 (4.16 %)	None	1 (4.76%)
Leg ulcer	2 (8.33 %)	1 (3.33 %)	2 (9.52 %)
Frequency of blood transfusion	9 (37.5 %)	13 (43.33%)	10 (47.61 %)

## References

[1] Garner C, Tatu T, Game L, Cardon LR, Spector TD, Farrall M (2000). A candidate gene study of F cell levels in sibling pairs using a joint linkage and association analysis. GeneScreen.

[2] Thein SL, Wainscoat JS, Sampietro M, Old JM, Cappellini D, Fiorelli G (1987). Association of thalassaemia intermedia with a β-globin gene haplotype. Br JHaematol.

[3] Labie D, Pagnier J, Lapoumeroulie C, Rouabhi F, Dunda-Belkhodja O, Chardin P (1985). Common haplotype dependency of high G y-globin gene expression and high Hb F levels in β thalassemia and sickle cell anemia patients. Proc Natl Acad Sci USA.

[4] Thein Swee Lay, Menzel Stephan, Lathrop Mark, Garner Chad (2009). Control of fetal hemoglobin: new insights emerging from genomics and clinical implications. Hum Mol Genet.

[5] Huisman THJ, Harris H, Gravely M, Schroeder WA, Shelton JR, Shelton JB, Evans L (1977). The chemical heterogeneity of the fetal hemoglobin in normal newborn infants and in adults. J Mol Cell Biochem.

[6] Gardiner MB, Reese AL, Headlee ME, Huisman THJ (1982). The heterogeneity of the gama chain of fetal hemoglobin in Hb S heterozygotes. Blood.

[7] Powars PA, Altay C, Huisman THJ, Smithies O (1984). Two novel arrangements of the human fetal globin genes: G-gama and A -gama-A gama. NucI Acids Res.

[8] Gilman JG, Huisman THJ (1984). Two independent genetic factors in the beta-globin gene cluster are associated with high G levels in the HbF of SS patients. Blood.

[9] Harano T, Reese AL, Ryan R, Abraham BL, Huisman THJ (1985). Five haplotypes in black beta -thalassaemia heterozygotes: Three are associated with high and two with low G values in fetal haemoglobin. Br J Haematol.

[10] Balgir RS (2000). The burden of haemoglobinopathies in India and the challenges ahead. Curr Sci.

[11] Verma IC (2000). Burden of genetic disorders in India. Indian J Pediatr.

[12] International Committee for Standardization in Haemotology (1978). Recommendations for selected methods for quantitative estimation of HbA2 and HbA2 reference preparation. Brit J Haemat.

[13] Gilman JG, Huisman THJ (1985). DNA sequence variation associated with elevated fetal Gg globin production. Blood.

[14] Sutton M, Bouhassira EE, Nagel RL (1989). Polymerase chain reaction amplification applied to the determination of beta like globin gene cluster haplotypes. Am J Hematol.

[15] Akinsheye I, Alsultan A, Solovieff N, Ngo D, Baldwin CT, Sebastiani P, Chui DVH, Steinberg MH (2011). Fetal hemoglobin in sickle cell anemia. Blood.

[16] Makani Julie, Menzel Stephan, Nkya Siana, Cox Sharon E, Drasar Emma, Soka Deogratius (2011). Genetics of fetal hemoglobin in Tanzanian and British patients with sickle cell anemia. Blood.

[17] Kato GJ, Gladwin MT, Steinberg MH (2007). Deconstructing sickle cell disease: Reappraisal of the role of hemolysis in the development of clinical subphenotypes. Blood Rev.

[18] Nolan VG, Adewoye A, Baldwin C (2006). Sickle cell leg ulcers: associations with haemolysis and SNPs in Klotho, TEK and genes of the TGF-beta/BMP pathway. Br J Haematol.

[19] Dedoussis GV, Mandilara GD, Boussiv M, Loutradis A (2000). HbF production in b-thalassaemia heterozygotes for the IVSII-1 G-A β0-globin mutation. Implication of the haplotype and the Gg-158 C-T mutation on the HbF level. Am J Hematol.

[20] Winichagoon P, Fucharoen S, Chen P, Wasi P (2000). Genetic factors affecting clinical severity in b-thalassaemia syndromes. J Pediatr Hematol Oncol.

[21] Kultar A, Kultar F, Wilson JB, Headlee MG, Huisman THJ (1984). Quantification of haemoglobin componenets by Highperformance cation exchange liquid chromatography. Am J hematol.

[22] Garner C, Tatu T, Reittie JE, Littlewood T, Darley J, Cervino S (2000). Genetic influenceson F cells and other hematologic variables: a twin heritability study. Blood.

[23] Serjeant GR, Serjeant GR (2001). Sickle cell- β thalassemia. Sickle Cell Disease.

[24] Panigrahi I, Agarwal S, Gupta T, Singhal P, Pradhan M (2005). Hemoglobin E-beta Thalassemia: Factors Affecting Phenotype. Indian pediatrics.

[25] Shimmoto MM, Vicari P, Fernandes AC, Guimaraes GS, Figueiredo MS (2006). XmnI polymorphism is associated with fetal hemoglobin levels in hypoplastic syndromes. Sao Paulo Med J.

[26] Oberoi S, Das R, Panigrahi I, Kaur J, Marwaha RK (2011). Xmn1-(G) γ polymorphism and clinical predictors of severity of disease in β-thalassemia intermedia. Pediatr Blood Cancer.

[27] Aditya Raina, Verma IC, Saxena Renu, Kaul Dinesh, Khanna VK (2006). Relation of Xmn-1 Polymorphism & Five Common Indian Mutations of Thalassaemia with Phenotypic Presentation in β-thalassaemia. JK Science.

[28] Embury SH, Dozy AM, Miller J, Davis JR, Kleman KM, Preisler H (1982). Concurrent sickle-cell anemia and thalassemia: Effect on severity of anemia. N Engl J Med.

[29] Schroeder WA, Powars DR, Kay LM (1989). β-cluster haplotypes, α-gene status and hematologic data from SS, SC, and Sβ-thalassemia patients in Southern California. Hemoglobin.

[30] Nemati Hooshang, Rahimi Zohreh, Bahrami Gholamreza (2010). The Xmn1 polymorphic site 5′ to the Gγ gene and its correlation to the Gγ:Aγ ratio, age at first blood transfusion and clinical features in β-Thalassemia patients from Western Iran. Mol Biol Rep.

